# Dirty Ends: Formation, Repair, and Biological Relevance of Non-Canonical DNA Terminal Structures

**DOI:** 10.3390/genes16101188

**Published:** 2025-10-13

**Authors:** Seanmory Sothy, Linlin Zhao

**Affiliations:** 1 Department of Chemistry, University of California, Riverside, Riverside, CA 92521-0403, USA; seanmory.sothy@email.ucr.edu; 2 Environmental Toxicology Graduate Program, University of California, Riverside, Riverside, CA 92521-0403, USA

**Keywords:** base excision repair, DNA damage, DNA repair intermediates, 2-deoxyribose oxidation, inflammation, immune signaling, nucleic acid modifications, mitochondrial DNA

## Abstract

Human DNA is continuously exposed to endogenous and exogenous agents that generate over 100,000 lesions per cell each day. In addition to damage to nucleobases, deoxyribose, and phosphate groups, a particularly harmful class of lesions involves non-canonical DNA termini—structures deviating from the canonical 3′-hydroxyl and 5′-phosphate ends. These aberrant DNA ends can obstruct essential DNA transactions and, if left unrepaired, contribute to cytotoxicity and mutagenesis. Their biological significance is further highlighted by the severe pathologies linked to deficiencies in DNA end-processing enzymes, including inflammation, cancer predisposition syndromes, neurodegeneration, and aging. This review highlights recent advances in our understanding of the formation, prevalence, and repair mechanisms of several key non-canonical DNA end structures, including 3′-phosphate, 3′-phosphoglycolate, 3′-α,β-unsaturated aldehyde and its glutathione derivative, 5′-deoxyribose-5-phosphate, 2′-deoxyribonucleoside-5′-aldehyde, and 5′-adenosine monophosphate. These non-canonical DNA terminal structures arise from various sources, such as radical-induced oxidation of the 2-deoxyribose moiety and DNA repair pathways. While this review does not cover the full spectrum of non-canonical termini, the selected structures are emphasized based on quantitative data supporting their biological relevance. The review also discusses their broader implications in mitochondrial DNA maintenance and inflammatory signaling and highlights key knowledge gaps that warrant further investigation.

## 1. Introduction

Genomic instability is a fundamental enabling factor for multiple hallmarks of cancer [1]. The maintenance of genome integrity is critically dependent on a variety of DNA repair genes; consequently, defects in these genes have been linked to cancer predisposition syndromes and immunological disorders [2]. While significant interest has focused on damage to nucleobases and the sugar backbone, emerging research demonstrates that some of the non-canonical DNA terminal structures—those other than the canonical 3′-hydroxyl (3′-OH) and 5′-phosphate (5′-P)—exist at significant levels. These structures, often called ‘dirty ends,’ can form during chemotherapy, radiotherapy, exposure to chemicals, and even as intermediates of DNA repair itself. Because they are incompatible with the enzymatic machinery of DNA synthesis and ligation, they pose tremendous threats to genomic stability [3,4,5].

The biological importance of efficiently cleansing these termini is underscored by the association between deficiencies in key DNA end-cleansing enzymes and various pathologies, including inflammation, cancer-predisposition syndromes, neurodegeneration, and aging [2]. For example, deficiencies in enzymes like tyrosyl-DNA phosphodiesterase 1 (TDP1) are linked to neurodegenerative diseases such as spinocerebellar ataxia with axonal neuropathy (SCAN1) [6,7]. Deficiency in polynucleotide kinase phosphatase (PNKP) in human cancer cells shows upregulation of interferon-stimulated genes even without genotoxic stress [8]. Moreover, mouse models with a gastric cancer-associated variant of pol β (L22P) that is defective in dRP-lyase activities exhibit chronic inflammation and tumor development [9,10,11]. In this review, we will discuss the major pathways leading to the formation of these non-canonical DNA termini, detail the enzymatic mechanisms responsible for their removal, and their relevance to human health.

**Figure 1:**
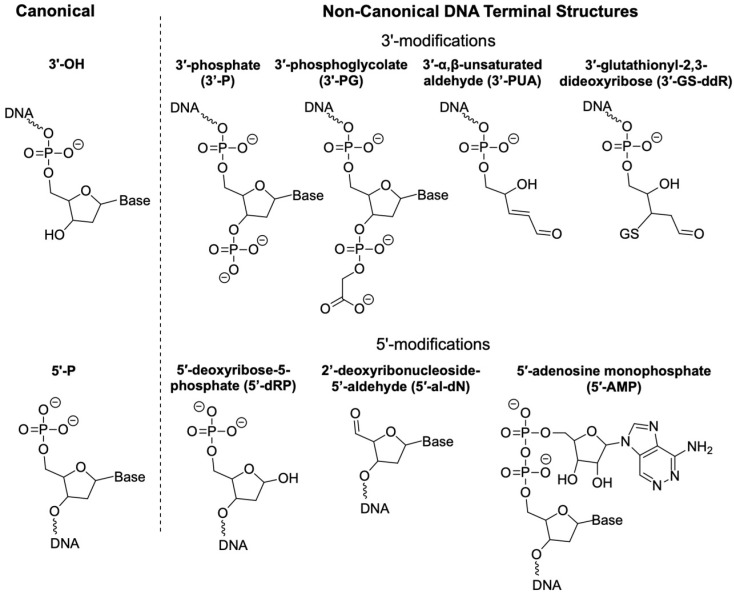
Selected non-canonical DNA terminal structures discussed in this review. 3′-phosphate (3′-P), 3′-phosphoglycolate (3′-PG), 3′-(α,β-unsaturated aldehyde) (3′-PUA), 3′-glutathionyl-2,3-dideoxyribose (3′-GS-ddR), 5′-deoxyribose-5-phosphate (5′-dRP), 2′-deoxyribonucleoside-5′-aldehyde (5′-al-dN), and 5′-adenosine monophosphate (5′-AMP).

This review focuses on several prevalent non-canonical DNA structures: 3′-phosphate (3′-P), 3′-phosphoglycolate (3′-PG), 3′-(α,β-unsaturated aldehyde) (3′-PUA) and its glutathione (GSH) conjugate (3′-GS-ddR), 5′-deoxyribose-5-phosphate (5′-dRP), 2′-deoxyribonucleoside-5′-aldehyde (5′-al-dN), and 5′-adenosine monophosphate (5′-AMP) (Figure 1). These lesions primarily derive from 2-deoxyribose oxidation or arise as intermediates during base excision repair (BER). They have been selected for discussion based on recent advancements in methods to measure their cellular levels and a growing understanding of their biological importance. While the enzymology of these repair pathways has been discussed in detail previously [3], this review aims to summarize the latest findings on their formation, repair, and biological implications. Other forms of heterogeneous DNA end structures, such as bulky DNA-protein cross-links (DPCs), have been reviewed elsewhere [12,13], and interested readers are referred to these excellent publications.

## 2. Non-Canonical DNA Structures

### 2.1. 3′-P

**Figure 2:**
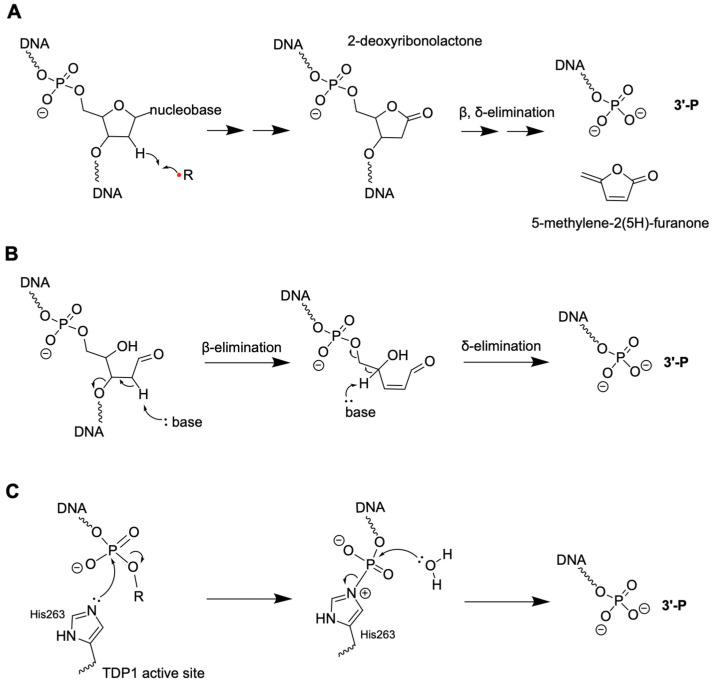
Three major pathways of 3′-P formation. (**A**) C1′ oxidation of 2-deoxyribose in DNA can lead to the formation of 2-deoxyribonolactone, followed by β,δ-elimination to yield 3′-P. (**B**) AP sites can undergo β,δ-elimination spontaneously under a base catalyst or enzymatically to form 3′-P. (**C**) TDP1-mediated DNA cleavage produces 3′-P. R represents a 3′-lesion immediately next to a nick or an AP site within an intact DNA strand. The red dot indicates an unpaired electron.

While the precise cellular level of 3′-P is not readily available in the literature, multiple lines of evidence indicate that it is a common DNA terminal modification under various genotoxic stress conditions. A major route involves 2-deoxyribose oxidation in DNA [3,14], initiated by hydrogen abstraction by radical species (e.g., hydroxyl radicals) or metal complexes (e.g., oxygen·Fe(II)·bleomycin complex or copper-phenanthroline complexes). Hydroxyl radicals can be sourced from radiolysis of water or endogenous metabolism [14,15,16,17,18,19]. Under biologically relevant conditions, all five positions in 2-deoxyribose in DNA can undergo oxidation, via hydrogen atom abstraction by radicals occurring at diffusion-controlled rates [14,20]. The reaction mechanisms have been discussed in previous reviews [14,15,20,21]. The reactivity of the various hydrogen atoms in 2-deoxyribose is governed by their solvent accessibility, occurring in the following order: 5′ > 4′ > 3′ ≈ 2′ ≈ 1′ [22]. The formation of 3′-P typically involves the elimination of labile intermediates formed in previous steps. For example, hydrogen abstraction at the 1′ position of 2-deoxyribose yields 2-deoxyribonolactone, which undergoes subsequent β, δ-elimination reactions to generate 3′-P (Figure 2A). The entire spectrum of oxidized 2-deoxyribose products and derivatives is quite complex, and their formation is affected by reaction conditions (e.g., aerobic vs. anaerobic) [23].

The second formation pathway involves β,δ-elimination at the abasic (AP) sites, which will be discussed in detail in the 3′-PUA section (Figure 2B) [24,25,26]. The third pathway involves enzymatic cleavage of DNA, such as TDP1-catalyzed DNA cleavage at lesions, such as at 3′-DPCs (e.g., topoisomerase 1 [7,27]) or AP sites (AP endonuclease activity [28]) to form 3′-P DNA termini (Figure 2C). As a member of the phospholipase D (PLD) enzyme superfamily, TDP1 uses His263 to attack the phosphate at the 5′ of the lesion (DPCs or AP sites), forming a transient TDP1-DNA complex at the active site (Figure 2C). Subsequently, a water molecule hydrolyzes the N–P bond of the TDP1-DNA intermediate, releasing the 3′-P [27]. Other enzymatic ways of 3′-P formation include deoxyribonuclease II (DNase II)-mediated degradation of exogenous DNA from phagocytosis [29,30].

Enzymes such as PNKP and AP endonuclease 1 (APE1) are known to remove 3′-P from DNA [3]. Mammalian PNKP is a bifunctional enzyme with 5′-kinase and 3′-phosphatase activities, involved in DNA repair pathways, such as BER, single-strand break repair, and non-homologous end-joining (NHEJ) [31,32,33,34]. Its kinase and phosphatase domains are explicitly built for this kind of interface DNA interaction and allow active site crosstalk [35,36]. While its phosphatase active site is highly effective for processing 3′-P, its specific structural requirements make it unsuitable for removing bulkier lesions like 3′-PG [37].

APE1 is a multifunctional enzyme in DNA repair, exhibiting AP endonuclease, 3′-phosphodiesterase, and 3′ to 5′ exonuclease activities [38,39]. The latter two activities are related to the removal of the 3′ terminal modifications. APE1 prefers dsDNA with nicks and has negligible activity on ssDNA 3′ ends, whereas PNKP acts on ssDNA and has poor activity on nicks (see below). While it was presumed that its 3′-phosphatase or exonuclease activities operate via a mechanism similar to its well-characterized endonuclease activity, recent crystallographic data have shown key differences between the two operating modes [40,41]. The major differences include (i) DNA bending and fraying of the 3′ end at the site of the nick and (ii) DNA intercalation by amino acid residues Arg177 and Met270. First, APE1 bends the DNA substrate similarly when binding to nicked substrates containing mismatched pairs or 8-oxo-7,8-dihydro-2′-deoxyguanosine (8-oxodG) at the 3′ end of the nick. Placing the 8-oxodG lesion at the 3′ end of the nick allows the assessment of the 3′ to 5′ exonuclease activity of APE1 [41]. By comparison, the bending is less when APE1 binds to an AP-containing substrate. Second, while Arg177 adopts similar conformations as a ‘space-filling’ residue, it interacts differently with opposite base residues (terminal dA:8-oxodG or dC:8-oxodG pairs) or a water molecule (AP sites) with different substrates [41]. An induced space-filling model has been proposed for APE1, whereby Arg177 and Met270 intercalate DNA to form a long and narrow binding pocket upon APE1-DNA binding [40]. The spatial arrangement at the active site seems to be able to explain the structural selectivity of the different DNA substrates (e.g., recessed or blunt-ended) with minimal sequence preference [40]. Notably, a change in the two intercalating residues in an APE1 ortholog (*Arabidopsis* apurinic endonuclease-redox protein) results in a DNA sequence (i.e., orphan base)-dependent activity with AP-containing dsDNA substrates and poor AP-DNA cleavage activities with ssDNA [42].

The biological importance of 3′-P has been implicated in cell lines with defective PNKP, the primary enzyme responsible for removing 3′-P from DNA. In addition to the nucleus, PNKP also localizes to mitochondria and is the major 3′-phosphatase in the organelle [43]. For example, PNKP knockdown compromises BER and SSBR activities in mitochondrial extracts of HEK293 cells [43]. PNKP-deficient HEK293 and A549 cells contain increased overall DNA lesion levels under both untreated and H_2_O_2_-treated conditions based on a qPCR-based DNA damage assay [43,44]. When complementing PNKP cells with constructs expressing wild-type, kinase-null, or phosphatase-null variants, only wild-type cells exhibit full repair capacity, suggesting that both kinase and phosphate activities are needed to maintain mtDNA integrity [44]. PNKP-depleted human cancer cells exhibit upregulation of interferon-stimulated genes, STAT1 phosphorylation, and accumulation of cytosolic DNA—markers of type I interferon response. The stimulation of type I interferon response is observed without treatment of exogenous chemicals and is likely due to compromised mtDNA repair abilities under decreased PNKP levels [8]. These observations are consistent with the important role of mtDNA in innate immunity and inflammatory responses [45,46]. Cumulatively, these results points toward the importance of DNA end-cleansing activities of the 3′-P end to generate a proper 3′-OH terminus.

### 2.2. 3′-PG

**Figure 3:**
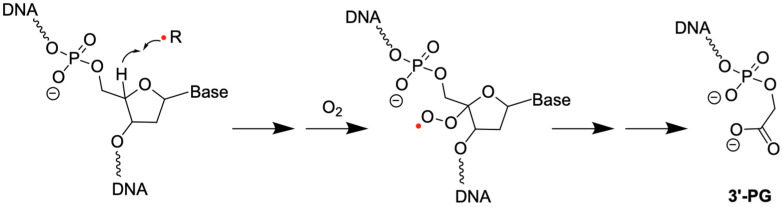
Formation of 3′-PG. An example of 2-deoxyribose oxidation via hydrogen abstraction at the C4′ position on the 2-deoxyribose, followed by the addition of O_2_ to form 3′-PG. The red dot indicates an unpaired electron.

3′-PG is another common terminal modification formed during 2-deoxyribose oxidation in DNA. It can be produced from the oxidation of the C4′ hydrogen on the 2-deoxyribose, followed by the addition of O_2_ (Figure 3) [20,47,48]. 3′-PG often coexists with 3′-P, with varying relative abundance depending on experimental conditions. For example, γ-radiation (high-energy photon, high penetration) under nitrogen generates almost exclusively 3′-P-containing DNA strand breaks, whereas irradiation under air increases the abundance of strand breaks by 3-fold, with a 3′-PG:3′-P ratio of 1.6:1 [49]. Furthermore, different radiation particles affect the formation of 3′-terminal modifications. For example, α-particle (two protons and two neutrons bound together, low penetration) is reported to generate 0.13-3′-PG per 10^6^ nucleotides (nt), while γ-radiation can generate 1.5-3′-PG per 10^6^ nucleotides [14,50,51], according to gas chromatography—mass spectrometry (GC-MS)-based quantification. Notably, 3′-PG is more stable than most of the non-canonical DNA terminal structures [52,53], implicating the need for enzymatic regulation of its levels. Because of its stability, 3′-PG has been used as a model 3′ end blocking lesion in various studies in identifying the enzymes for removing 3′ lesions.

Indeed, multiple studies have demonstrated the important roles of APE1 and TDP1 in removing 3′-PG lesions. Recombinant APE1 can process dsDNA substrates containing a 3′-PG lesion at the nick in vitro [54]. In whole cell extracts of HeLa cells, the removal of 3′-PG from double-stranded (ds) DNA substrates is dependent on APE1, as evidenced by immunodepletion experiments [55]. The efficiency of APE1 is dependent on the substrate structure, with the internal gapped substrate being the most efficiently processed. Nearly no activity was observed on a dsDNA substrate bearing a 1 to 2-nt overhang with a 3′-PG lesion [56]. On the other hand, substrates containing 3′-PG lesions at double-strand breaks can be processed by recombinant TDP1 and whole cell extracts [57]. By contrast, cell extracts from neurodegenerative disease SCAN1 patients, who have a homozygous mutation (H493R) in the active site of TDP1 [6], cannot process the same substrate [58]. Notably, the TDP1-processed product yields 3′-P, which would require PNKP for further processing. Furthermore, Artemis nuclease, an enzyme involved in V(D)J recombination, can process dsDNA substrates containing 3′-PG [59]. Artemis efficiently cleaves the overhang portion of dsDNA substrates bearing a 3′-PG lesion, and such activities depend on DNA-dependent protein kinase and ATP and can be stimulated by Ku [59]. Lastly, a structure-specific endonuclease, XPF-ERCC1, has been shown to cleave 3′-PG-containing DNA fragments in vitro [60]. XPF-ERCC1 is essential for nucleotide excision repair and DNA interstrand cross-link repair. These studies highlight the roles of different repair pathways and enzymes in guarding the genome from 3′-PG accumulation.

### 2.3. 3′-PUA and 3′-GS-ddR

As abundant endogenous DNA lesions and key repair intermediates, AP sites can form during spontaneous depurination/depyrimidination and BER. AP sites can undergo β-elimination to form 3′-PUA, with a faster rate in the presence of amine catalysts like biological polyamines, such as spermine and spermidine [61]. 3′-PUA is also a repair intermediate by bifunctional DNA glycosylases, which cleave the damaged nucleobase and then the DNA backbone at the AP lesion. While it is believed that BER operates in a “substrate channeling” model to avoid exposure of toxic repair intermediates to the cellular environment, direct experimental evidence supporting the model remains scarce. In fact, a study on the mouse embryonic stem cell suggested that 3′-PUA is present at around 1.7 lesions per 10^6^ nt based on liquid chromatograph–tandem mass spectrometry (LC-MS/MS) experiments [62].

**Figure 4:**
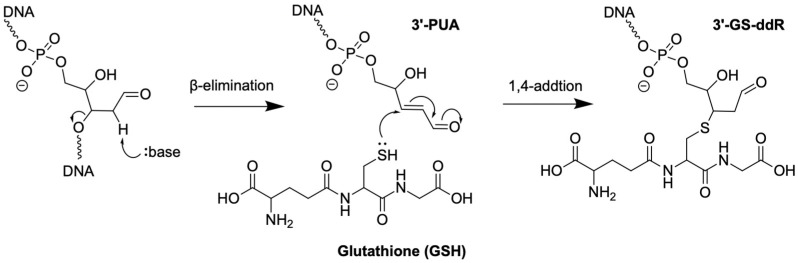
Formation of 3′-PUA and 3′-GS-ddR.

The chemical reactivity of 3′-PUA allows the formation of additional derivatives. For example, 3′-PUA has also been shown to form DPCs with Poly (ADP-ribose) polymerase 1 (PARP1) protein [63], human AlkB homolog 1 (ALKBH1) [64], and mitochondrial transcription factor A (TFAM) [65]. In addition, biological thiol GSH is present at millimolar concentrations, and thiol conjugation to α, β-unsaturated aldehyde is both kinetically and thermodynamically favorable (Figure 4) [66]. It is well documented that conjugation with thiols represents a crucial detoxification pathway for α, β-unsaturated aldehydes formed in various metabolic processes [67,68]. In the context of reactions with DNA, Bailly and Verly first reported that AP site-containing DNA can form thiol-derivatives in the presence of spermine and 2-mercaptoethanol based on gel electrophoretic analysis [69]. However, it was not until recently that Gates and colleagues unequivocally demonstrated the formation of 3′-GS-ddR from reactions of AP sites and GSH in the presence of spermine [70,71]. Notably, 3′-GS-ddR is relatively unstable (*t*_1/2_~6 h) in the presence of physiological levels of spermine; however, its stability significantly improves in the presence of 5 mM GSH at pH 7.4 (stable for 60 h), suggesting its potential accumulation in the genome [70]. Using mass spectrometry-based quantification, Wang and colleagues obtained a level of 2−5 GS-ddR per 10^7^ nucleosides in total cellular DNA of HEK293T cells upon exposure to an alkylating agent, *N*-methyl-*N*-nitrosourea (MNU) [71]. Notably, GS-ddR is 20 to 80-fold higher in abundance in mitochondrial DNA compared to nuclear DNA, and its accumulation is associated with the downregulation of several ribosomal and complex I subunit proteins and the upregulation of proteins related to redox balance and mitochondrial dynamics [72], implicating its roles in mitochondrial genomic instability or immune signaling.

Given the documented activities of APE1 in removing the 3′ lesions, it is not surprising that 3′-GS-ddR are subject to APE1 excision in vitro [70]. In HEK293T cells, treating cells with an APE1 inhibitor led to a greater extent of GS-ddR lesion accumulation [71]. It is reasonable to speculate that TDP1 can process 3′-GS-ddR lesions due to its ability to cleave various peptide-DNA conjugates at the 3′-end of DNA substrates in vitro [73]. Indeed, it has been shown that both APE1 and TDP1 can remove 3′-GS-ddR lesions in vitro and in mtDNA in HEK293A cells [72]. Given their different substrate preference, it has been proposed that APE1 plays a major role in regulating the mitochondrial 3′-GS-ddR lesions under alkylation DNA damage by *N*-methyl-*N*-nitrosourea, whereas TDP1 is more important without genotoxic stress [72]. The specific roles of these repair enzymes in removing 3′-PUA and 3′-GS-ddR under different genotoxic stress conditions remain to be firmly established.

### 2.4. 5′-dRP

In BER, 5′-dRP is formed as a repair intermediate of AP sites when APE1 cleaves the phosphodiester bond immediately 5′ to the AP site. Subsequently, 5′-dRP is removed by the dRP-lyase activity of DNA polymerase (pol) β [74] or possibly pol λ [75], followed by gap-filling synthesis and ligation to complete the repair process. While it is commonly believed that DNA repair intermediates are sequestered by repair enzymes [76,77,78,79], experimental evidence from biological systems to conclusively confirm this is lacking. Recent single-molecule studies using recombinant enzymes indicate that a significant fraction of DNA-binding events by APE1 and pol β do not always facilitate substrate channeling [80], suggesting the potential of 5′-dRP accumulation. In addition, 5′-dRP can form during chemotherapy, radiotherapy, or chronic inflammation [3,4,5].

5′-dRP can be processed by the lyase activity of several enzymes, such as pol β [74], pol λ [75], pol ι [81], pol θ [82], and Ku [83]. Here, we focus on pol β as a prototypical dRP lyase and discuss the mechanism and importance of the activity. Pol β belongs to the X-family of DNA polymerases that also include pol λ, pol μ, and terminal transferase [84]. Pol β contains an 8-kDa lyase domain and a 31-kDa polymerase domain. The lyase domain controls the DNA binding affinity to DNA substrates containing a 1-nt gap, as evidenced in mutagenesis and cellular experiments [85]. The importance of the pol β lyase domain in gapped substrate recognition is further supported by cryo-EM structures of nucleosome core particle-DNA-pol β complexes [86]. These structures demonstrate that the lyase domain mediates the initial nucleosome binding through interacting with the 5′ phosphate group. Mechanistically, the three lysine residues at the lyase active site confer the specificity to the DNA substrate with a 1-nt gap and destabilize nonspecific DNA binding [85]. Time-resolved X-ray crystal structures also demonstrate that the lyase enhanced the DNA polymerase activity after the formation of a transient covalent cross-link with 5′-dRP-containing DNA fragment using K72 at the lyase active site [87]. These studies have provided mechanistic insights into the role of the lyase domain in the overall end-cleansing and gap-filling repair processes.

Alternatively, 5′-dRP can also be processed by strand displacement DNA synthesis followed by 5′-dRP-DNA flap removal (e.g., by FEN1). The alternative pathway can occur in the presence of a 2-deoxyribonolactone [88,89] or reduced abasic lesion [90], whereby pol β is unable to remove the sugar residue. The repair mechanism is a subpathway of mammalian BER, known as long-patch BER, with the other being single-nucleotide BER [76]. In vitro assays using cell extracts and synthetic DNA substrates have demonstrated that the two pathways occur at approximately equal frequency [91]. The exact mechanism of the subpathway choice is an active area of research.

Because BER is a major DNA repair pathway in mitochondria, several mitochondrial proteins have also been investigated for their dRP lyase activities. For example, the major mitochondrial pol γ has dRP lyase activities in vitro [92]. By comparison, the catalytic efficiency of pol γ is approximately 20-fold lower than that of pol β in dRP lyase reactions [92]. In this context, pol β has been shown to localize to mitochondria in certain tissues [93,94]. Further, the major DNA packaging protein mitochondrial transcription factor A (TFAM) possesses dRP lyase activities in vitro [95]. The functional roles of the dRP lyase activities of these proteins in mitochondria remain to be clarified in cells and in vivo. Besides BER, the 5′-dRP removal is part of SSBR, likely due to many shared enzymes with BER [96]. Enzymes with 5′-dRP lyase activities, such as pol λ, pol θ, and Ku, are involved in non-homologous end joining (NHEJ) [83] and microhomology-mediated end-joining (MMEJ) [97].

Directly characterizing 5′-dRP remains challenging due to its inherent chemical lability and reactivity. 5′-dRP residues undergo spontaneous β-elimination, with a reported half-life of only 2 to 3 h [98,99,100], which has hindered the reliable characterization. Simonelli et al. used site-specific modified shuttle vectors followed by transfection into monkey COS7 cells and quantitative PCR to analyze the mutagenic effects of AP sites, 5′-dRP, and 3′-PUA [101]. According to their results, all three lesions led to deletion mutations, with a frequency of 9% for AP sites, 16% for 5′-dRP, and approximately 30% for 3′-PUA. The remaining mutations were point mutations with a strong preference for dAMP insertions opposite all three lesions. For 5′-dRP, dGMP was also incorporated at a frequency similar to dAMP opposite the lesion. The short half-life of 5′-dRP and potential reactions of 3′-PUA with cellular thiols raise the question of whether the lesions on modified vectors retain their true chemical identity. In addition, the results do not account for cell death, as the authors acknowledged. Furthermore, its shared aldehyde functional group makes it chemically indistinguishable from AP sites. For example, a common aldehyde-reactive probe, ARP (*N*-(aminooxyacetyl)-*N*′-biotinylhydrazine), reacts non-selectively with both lesions. Therefore, the exact 5′-dRP levels and biological roles under various genotoxic stress conditions remain to be clarified.

Nonetheless, the importance of 5′-dRP removal has been implicated in phenotypes in cell lines and animal models with a deficiency in enzymes with dRP-lyase activities. For example, the dRP-lyase activity of pol β is essential for reversing hypersensitivity to methylating agents or the anticancer drug temozolomide in pol β-null mouse fibroblasts [102,103]. The activity also contributes to temozolomide resistance in glioma cells [104]. Conditional knockout mice expressing a dRP-lyase-deficient pol β variant (L22P, a gastric cancer-associated variant) exhibit hyperproliferation, increased DNA DSBs, cytosolic DNA-mediated inflammation, and stomach tumors [9,10,11]. Clearly, further investigation into the functional importance of 5′-dRP in inflammation and cancer etiology is warranted.

### 2.5. 5′-al-dN

**Figure 5:**
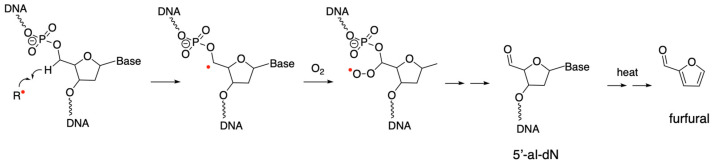
Formation of 5′-al-dN. Hydrogen abstraction at the C5′ position, followed by peroxyl radical formation, leads to the formation of 5′-al-dN. 5′-al-dN is unstable and often converted to furfural (released from DNA) under heating and alkali conditions to induce β,δ-elimination. The red dot indicates an unpaired electron.

As discussed above, the C5′ hydrogen is most susceptible to deoxyribose oxidation due to solvent accessibility [22]. The C5′ radical is the most common 2-deoxyribosyl radical formed by direct ionization of the sugar−phosphate backbone [105]. At physiological concentrations, the C5′ radical readily converts to the corresponding C5′ peroxyl radical, followed by the formation of 5′-al-dN (Figure 5) [14]. Neocarzinostatin and other selective reagents are known to form 5′-al-dN as the major product when damaging DNA [106,107]. Furthermore, under physiological conditions, 5′-al-dN is relatively stable, with a half-life of a few days [108,109]. The stability can increase, decrease, or remain unchanged depending on its location within reconstituted nucleosome core particles [109]. This is different from a general decreasing trend in the half-life of AP sites in nucleosome core particles compared to free DNA [109].

Exploiting the lability of 5′-al-dN under alkaline conditions, several studies have used its conversion to furfural (released from DNA under heating and alkali conditions) as a proxy to quantify the lesion [110,111]. Using an isotope-dilution GC−MS method, Chan et al. quantified furfural and 5-methylene-2(5H)-furanone (5MF), as a proxy for two major 2-deoxyribose oxidation products, 5′-al-dN and 2-deoxyribonolactone abasic lesion, respectively [111]. In TK6 human lymphoblastoid cells, 5′-al-dN forms at a level of 2.2 lesions per 10^7^ nucleosides per Gy (G-value 74 nmol/J) and 2-deoxyribonolactone abasic lesion at 0.45 lesions per 10^7^ nucleosides under per Gy γ-irradiation conditions [111]. The relative abundance of the two lesions is consistent with the solvent accessibility being the major factor in 2-deoxyribose oxidation [22]. A recent study compared the level of 5′-al-dN to that of a commonly used oxidative DNA damage marker, 8-oxodG [112]. When a synthetic DNA substrate is exposed to a low dose of ionizing radiation (100 Gy), 5′-al-dA and 5′-al-dG are the two prominent 5′-al-dN lesions forming at a level of approximately 1/3 of the 8-oxodG. Notably, the study also identified a novel 5′-carboxylate product, 5′-carboxylate dN, formed at approximately ¼ of the corresponding 5′-al-dN [112], highlighting the complexity of radical-mediated DNA oxidation and the need for characterizing additional lesions. The level of the 5′-carboxylate dN lesion remains to be established in cultured cells and biological samples.

In vitro, 5′-al-dN can be repaired by strand displacement synthesis of pol β, followed by flap removal by flap-endonuclease 1 (FEN1) [113,114], which is also known as long-patch BER. The repair process is not specific to 5′-al-dN but can occur with other 5′-end blocking lesions. Because of the relatively long half-life, 5′-al-dN has the potential to accumulate in the genome. Their genomic location and biological impact remain to be firmly established.

### 2.6. 5′-AMP

The DNA repair process is a multi-step process, with DNA ligation being the last step. DNA ligation involves three steps: (i) adenylation of the lysine residue in the ligase active site, (ii) transfer of adenosine monophosphate (AMP) moiety to activate the 5′-phosphate of the DNA, and (iii) the formation of the phosphodiester bond and release of 5′-AMP. Although the second and third steps occur in a concerted manner, a DNA ligase can dissociate prematurely, leading to the formation of an abortive intermediate 5′-AMP [115].

While the exact levels of 5′-AMP in the genome remain unknown, its biological importance is implicated in diseases associated with defective enzymatic activities in removing 5′-AMP. Ataxia oculomotor apraxia-1 (AOA1) is a rare and inherited neurological disorder caused by mutations in the gene (APTX) encoding aprataxin [116,117]. Aprataxin acts as a nucleotide hydrolase and transferase that specifically removes this 5′-AMP moiety, restoring a ligatable DNA end [115]. This function is critical for maintaining genome stability. Cells with defective aprataxin are sensitive to agents that cause DNA single-strand breaks and show elevated levels of chromosomal abnormalities [118,119,120].

Besides maintaining the nuclear genome, aprataxin also localizes to mitochondria. Knocking down of aprataxin in human SH-SY5Y neuroblastoma cells or primary skeletal muscle myoblasts results in lower mitochondrial activities (reflected in citrate synthase activity) and mtDNA copy number [121]. The overall capacity for removing 5′-AMP appears to be lower in mitochondrial extracts compared to nuclear extracts from APTX-deficient cells [122]. Depletion of APTX led to decreased expression of the mitochondrial inner membrane fusion protein optic atrophy type 1 (OPA1) protein that is crucial for mitochondrial fusion [123]. Furthermore, aprataxin plays a role in the immune system. APTX knockout microglial cells (immune cells of the brain) exhibit down-regulated cGAS-STING and RIG-I/MAVS pathways [124], which are responsible for sensing DNA and RNA viruses. Similarly, defective immune responses and dysregulated DNA- and RNA-sensing pathways were also observed in AOA1 patient-derived cell lines based on RNA-Seq data [124]. Clearly, the functions of aprataxin are not limited to DNA maintenance but also involve broader cellular processes and immune responses. Future research is needed to fully understand the intricate crosstalk between these pathways and explore their potential for therapeutic intervention.

## 3. Conclusions

This review has detailed the formation, repair, and biological implications of several non-canonical DNA structures that arise from genotoxic stress, particularly the oxidation of the 2-deoxyribose moiety. Lesions such as 3′-P, 3′-PG, 3′-PUA, 3′-GS-ddR, 5′-dRP, 5′-al-dN, and 5′-AMP represent a significant challenge to genome integrity. Their formation stems from diverse sources, including radical-induced hydrogen abstraction, the processing of AP sites, and enzymatic processes like TDP1. While chemically distinct, these lesions often block DNA replication and transcription, necessitating their efficient removal.

A recurring theme is the overlapping and multifaceted nature of the cellular repair machinery. Key enzymes, including APE1, PNKP, and TDP1, demonstrate broad substrate specificity, acting on multiple types of terminal modifications to restore canonical 3′-OH or 5′-P ends. A summary of the major enzymes discussed in this review is shown in Table 1. For instance, APE1 can process 3′-P, 3′-PG, and 3′-GS-ddR, while TDP1 also targets 3′-PG and is implicated in removing other complex 3′ adducts. The central role of PNKP as the primary 3′-phosphatase is highlighted by the severe cellular defects observed in its absence, linking the accumulation of 3′-P ends to compromised mitochondrial DNA integrity and the activation of inflammatory and innate immune responses. Similarly, deficiencies in the dRP-lyase activity of pol β, which is critical for removing 5′-dRP, lead to genomic instability, inflammation, and tumorigenesis. Collectively, this evidence underscores that the efficient cleansing of these non-canonical DNA termini is not merely a housekeeping function but a critical process for preventing neurodegeneration, cancer, and aberrant immune activation.

## 4. Future Perspectives

While significant progress has been made in identifying the key lesions and repair enzymes, several areas warrant further investigation to fully understand the biological impact of these non-canonical DNA structures. First, a major challenge is the difficulty in accurately quantifying these lesions in vivo, particularly chemically labile intermediates like 5′-dRP. The development of more robust and specific analytical techniques, which can distinguish between structurally similar lesions (e.g., 5′-dRP and AP sites), is crucial to establishing their exact levels under physiological and pathological conditions. Second, while in vitro studies have identified multiple enzymes capable of processing a single lesion (e.g., APE1, TDP1, and Artemis for 3′-PG), their division of labor and hierarchy within the cell remain unclear. Future research should focus on clarifying which pathways are dominant for specific lesions under different types of genotoxic stress, in different cell and tissue types, and within different subcellular compartments, such as the mitochondria versus the nucleus. Third, the recent identification of novel lesions, such as 5′-carboxylate dN, highlights that our understanding of DNA oxidation chemistry is incomplete. Future work should aim to characterize the formation, stability, and repair of these newly discovered lesions and assess their biological consequences. Last but not least, the link between the accumulation of 3′-P ends in PNKP-deficient cells and the stimulation of a type I interferon response points to a direct connection between DNA end structure and innate immune signaling. Further investigation is warranted to understand the precise mechanisms by which specific unrepaired DNA termini are sensed as “foreign” or “damaged” DNA species and lead to the activation of inflammatory pathways. Elucidating these connections will be critical for understanding the role of DNA damage in chronic inflammation, cancer etiology, and autoimmune diseases.

## Figures and Tables

**Table 1:** Major enzymes discussed in this review.

Enzyme Name (Gene)	Substrate	Product	Activity	Biological Relevance *
tyrosyl-DNA phosphodiesterase 1 (*TDP1*)	AP sites, 3′-DPCs, 3′-PG, 3′-PUA, 3′-GS-ddR	3′-P	AP endonuclease, phosphodiesterase, limited 3′-exonuclease	spinocerebellar ataxia with axonal neuropathy (SCAN1)
deoxyribonuclease II (*DNASE2*)	DNA, RNA	3′-P	endonuclease	autoinflammatory-pancytopenia syndrome and dyskeratosis congenita, autosomal dominant 6
polynucleotide kinase phosphatase (*PNKP*)	3′-P, 3′-OH	3′-OH, 3′-P	5′-kinase, 3′-phosphatase	Alzheimer’s, microcephaly, seizures, developmental delay, ataxia-ocular motor apraxia 4 (AOA4)
AP endonuclease 1 (*APEX1*)	AP sites, 3′-P, 3′-PG, 3′-PUA, 3′-GS-ddR	5′-dRP (from AP sites) and 3′-OH (from 3′-lesions)	AP endonuclease, 3′-phosphodiesterase, 3′-5′-exonuclease	malignancies and neurodegenerative diseases
Artemis nuclease (*DCLRE1C*)	3′-PG	3′-OH	exonuclease, endonuclease	Severe combined immunodeficiency with sensitivity to ionizing radiation, Omenn syndrome
DNA polymerase β (POLB)	5′-dRP	5′-P	dRP lyase, DNA polymerase	Werner syndrome, esophageal cancer
DNA Polymerase λ (*POLL*)	5′-dRP	5′-P	dRP lyase, DNA polymerase	Adams-Oliver syndrome, Xeroderma Pigmentosum, variant type

* Data from https://www.genecards.org (accessed on 30 September 2025).

## Data Availability

No new data were created or analyzed in this study.
